# Research on soil bacterial community assembly and function under different straw returning practices in arid and semi-arid agricultural ecosystems over multiple years

**DOI:** 10.3389/fmicb.2025.1590686

**Published:** 2025-05-12

**Authors:** Rui-Zhi Liu, Xiao-Ya Zhao, Biao Feng, Wen-Shan Zhao, Ming-Yu Li, Xiao-Fang Yu, Shu-Ping Hu, Rui-Ping Li, Ju-Lin Gao, Qinggeer Borjigin

**Affiliations:** ^1^College of Agriculture, Inner Mongolia Agricultural University, Hohhot, China; ^2^Key Laboratory of Crop Cultivation and Genetic Improvement of Inner Mongolia Autonomous Region, Hohhot, China; ^3^Inner Mongolia Autonomous Region Engineering Research Centre of Microorganisms for In Situ Corn Straw Return, Hohhot, China; ^4^Vocational and Technical College, Inner Mongolia Agricultural University, Baotou, China

**Keywords:** straw return practices, soil microbial community assembly, bacterial lifestyle, ecosystem stability, semi-arid agriculture

## Abstract

**Introduction:**

Straw return has gained attention for its potential to improve soil quality and crop yields, particularly in semi-arid regions like the Tumu Chuan Plain Irrigation Area. Soil bacteria play a crucial role in regulating soil biological processes, and understanding how straw return affects bacterial populations can guide better agricultural management practices.

**Methods:**

We investigated the impact of continuous straw return on soil bacterial communities using 16S rRNA gene sequencing. Four treatments were applied: Farmers’ shallow rotation (CK), straw incorporated with deep tillage (DPR), straw incorporated with subsoiling (SSR), and no-tillage mulching straw return (NTR). Bacterial community structure, metabolic pathways, and assembly mechanisms were analyzed using Bugbase and PICRUSt2 for phenotypic and metabolic pathway predictions.

**Results:**

The study found that straw return practices significantly altered the relative abundance and life history strategies of bacterial phyla, mainly influenced by soil organic matter (SOM) and enzyme activity. The K-strategist to r-strategist ratio was highest in CK (2.06) and lowest in SSR (1.89). DPR and NTR treatments significantly changed bacterial community structure compared to CK (*p* < 0.05), resembling SSR. Predictions showed that DPR and NTR enhanced carbohydrate and amino acid metabolism and promoted more stable bacterial networks, with homogenous selection and drift effects. Bacterial aggregation in all treatments was driven by random processes, with varying aggregation levels: CK (20%), DPR (38.6%), SSR (16.5%), and NTR (30.7%).

**Discussion:**

The study demonstrates that continuous straw return practices significantly impact soil bacterial communities. DPR and NTR notably improved microbial diversity, bacterial cooperation, and ecosystem stability. These findings provide valuable insights for sustainable agricultural practices in semi-arid regions, enhancing soil microbial ecology and soil health through strategic straw return.

## 1 Introduction

Approximately 40% of the world’s land area is located in arid or semi-arid regions, which are home to approximately one-third of the global agricultural population resides (Bernardino et al., 2025). These areas are crucial for global agricultural production but face challenges such as water scarcity, soil degradation, and environmental deterioration, which significantly limit the sustainability of agriculture. With increasing climate change and growing land resource pressures, enhancing agricultural productivity, restoring soil functions, and achieving ecological sustainability have become critical topics in current agricultural research (Yu et al., 2024). Straw return, as an effective agricultural management measure, has been widely used to improve soil physical structure, enhance water retention capacity, and promote nutrient cycling, which is key to improving soil quality and crop yield (Liu Q. et al., 2024). However, the long-term effects of different straw return practices on soil microbial communities, especially soil bacterial communities, remain unclear, particularly in dry and semi-arid regions where the effectiveness of straw return is influenced by various environmental factors.

Soil microorganisms, particularly bacteria, play a vital role in soil ecosystems by participating in organic matter decomposition, nutrient cycling, and maintaining soil health (Wang et al., 2024). Diverse tillage practices combined with straw return can regulate microbial dynamics in continuous cropping fields, thereby promoting crop nutrient uptake and productivity (Wang et al., 2025). Previous studies have shown that straw return provides a rich carbon sources for microbial communities, enhancing microbial activity and diversity (Liu et al., 2025). However, the mechanisms by which different straw return practices (e.g., deep plowing, no-tillage covering, straw composting) affect microbial communities are not well understood, particularly with respect to their long-term impact on soil health (Zhang J. et al., 2024). Straw direct covering promotes short-term microbial activity by providing labile organic matter, whereas deep plowing improves soil structure and long-term organic matter stability, ultimately influencing microbial community composition and function (Song et al., 2024). These changes may affect microbial ecological functions, such as nitrogen-fixing bacteria abundance or the microbial populations involved in cellulose and lignin decomposition. Microbial community assembly is driven by a combination of environmental selection, crop interactions, and random processes (Tian et al., 2024). Compared to non-straw return, straw return not only changes microbial community structure but also increases soil microbial resistance (Hao et al., 2019). The response of soil microbial communities to drought varies significantly among different straw return practices. In arid regions of northern China, straw return has been shown to significantly alter the complexity and stability of microbial symbiotic networks (Kang et al., 2025). Straw return, by improving soil health and microbial-host compatibility, positively impacts root-soil-microbe interactions and drought-resistant crops (Liu X. et al., 2023). Existing studies have primarily focused on the short-term effects of straw return on microbial community composition and diversity, with a lack of systematic analysis of the combined and long-term effects of different straw return practices on microbial community structure. Microbial responses to environmental selection affect life history strategies and community assembly processes, playing key roles in natural resource utilization and interspecies interactions (Kinnunen et al., 2020; Jiao et al., 2022; Yang et al., 2023), this response can, in turn, influence agricultural productivity under various straw return practices. However, the ecological function of soil bacterial communities, their adaptability to environmental stress, and their interspecies interactions in multi-year straw return practices in semi-arid/arid systems remain inadequately explored.

Maize is an important food crop (Zong et al., 2024), and straw return has significant effects on its yield and soil health (Liu R.-Z. et al., 2024; Chen et al., 2025). Previous studies have shown that straw return can alter the composition and function of soil microbial communities and enhance the activity of beneficial microbes (Yang et al., 2021). This study investigates on the long-term regulation of soil bacterial communities under different straw return practices in a continuous maize cropping system on the Tumu Chuan Plain Irrigation Area. The aim is to explore the effects of continuous straw return practices on bacterial community assembly mechanisms and functional traits, and to investigate the feedback relationships between straw return and soil microbial communities. Our hypotheses are: (1) continuous different straw return practices regulate bacterial community composition, structure, and life history strategies by altering soil physical and chemical properties and microbial habitats, (2) continuous deep plowing with straw incorporation and no-tillage straw covering enhance bacterial adaptation to environmental stress by improving soil conditions. By deeply analyzing the long-term effects of different straw return practices, this study provides scientific evidence for straw management in semi-arid and arid regions and contributes to the sustainable development of agricultural ecosystems.

## 2 Materials and methods

### 2.1 Experimental site

This study is based on the pre-constructed straw return trial platform of the group (the straw returning platform test site was established in 2018) conducted in 2020–2024 and was carried out in the China Chilechuan Modern Agricultural Expo Park (Beizhitu Village, Goumen Town, Tumet Right Banner, Baotou City, Inner Mongolia, latitude 40°28′28″N, longitude 110°29′5″E), where perennial straw return to the experimental field has been implemented starting in 2018. This area has a semi-arid mesothermal temperate continental monsoon climate, with an average annual temperature of 6–8°C, 400 mm of annual precipitation, a frost-free period of 140 days, an elevation of 1,015 m, 2,806 h of annual sunshine, and an annual active cumulative temperature ranging from 3,000–3,500°C. The site is used for continuous maize cultivation. In the absence of tillage, the soil texture is sandy loam, and the soil fertility is characterized by an organic matter content of 22.04 g/kg, an alkali-hydrolysable nitrogen content of 57.82 mg/kg, an available phosphorus content of 3.57 mg/kg, and an available potassium content of 84.97 mg/kg. The soil nutrient data collected before sowing and tillage (0–45 cm soil layer) are shown in Supplementary Table S1, and the main meteorological data collected during the test period are shown in Supplementary Figure S1.

### 2.2 Experimental design

The experiment used a one-factor experimental design, where the plowing method was applied in the central zone. Farmers’ shallow rotation (CK): refers to the sowing method used by local farmers. During the fall land preparation phase, the straw is crushed and baled, then removed from the field. In spring, after the stubble is processed, conventional shallow rotation sowing is carried out. Three treatments were established for comparison, straw incorporated with deep tillage (DPR): in autumn, straw is shredded and plowed into the soil to a depth of 30–40 cm. In spring, sowing is done using a conventional planter. Straw incorporated with subsoiling (SSR): in autumn, straw is shredded and mixed with the soil after subsoiling to a depth of 35–40 cm. In spring, sowing is done using a conventional planter. No-tillage mulching straw return (NTR): in autumn, straw is shredded and left on the soil surface as mulch. In spring, sowing is done using a no-till planter. All straw return treatments included full corn straw return at 135–150 kg ha^−1^. The maize variety planted was Xianyu 696 at a planting density of 825 plants/ha. Ammonium dihydrogen phosphate (N 18%, P 46%) was applied at a rate of 375 kg ha^−1^, and potassium sulfate (K 22%) was applied at a rate of 150 kg ha^−1^. Urea (N 46%) was applied utilized as a supplementary fertilizer with application rates of 30% at V6 (sixth leaf), 60% at V12 (twelfth leaf), and 10% at R2 (blister), resulting in a total nitrogen application of 345 kg/ha. Drip irrigation was performed four times during the growing season: at V6, V12, R1 (silking), and R2. Each irrigation event covered 750 m^3^/ha. All remaining management practices followed standard procedures commonly used in large-scale agricultural production.

The experiment was conducted annually during the maize pre-sowing period from 2020 to 2024. Soil samples were collected each May, totaling four sampling events. In each experimental plot, soil was collected from the 0–45 cm layer using an imported auger in an “S”-shaped sampling pattern. A total of 12 subsamples were taken per plot, thoroughly mixed, and passed through a 2 mm mesh sieve to remove plant debris, root fragments, and other impurities. The homogenized soil was then subjected to the quartering method to obtain a representative sample, sealed in sterile bags, and immediately transported to the laboratory. Each composite sample was divided into three parts: the first portion was air-dried for determining soil physicochemical properties and measuring the activity of alkaline phosphatase (ALP) (Tan et al., 2017; Wang et al., 2017) and catalase, the latter being assessed by back-titration of residual H₂O₂ with KMnO₄ (Johnson and Temple, 1964). The second portion was stored at 4°C for analysis of glutamine synthetase (Glu) activity (Haghighat, 2005), the third portion was stored at −80°C for DNA extraction and high-throughput sequencing.

### 2.3 Soil properties analysis

Soil Moisture (SM,%) was measured using the JL-01 multi-point soil temperature and humidity recorder (JL-01, Jingyi Electronic Company). Soil Bulk Density (g/cm^3^, BD) was determined by the core method (VanRemortel and Shields, 1993). Soil alkaline nitrogen (AN) was measured using the alkaline hydrolysis diffusion method (Bremner, 1960). Available phosphorus (AP) was determined with the Smartchem140 automated chemical analyzer (SMARTCHEM450, AMS, France) (Olsen and Sommers, 1982). Available potassium (AK) was measured using a flame photometer (M410, Sherwood Scientific, United States) (Allen and Grimshaw, 1986). Soil organic matter (SOM) was determined by the potassium dichromate method (Walkley and Black, 1934). Alkaline phosphatase (ALP) was determined by the phenylphosphate colorimetric method (Liu et al., 2020). Hydrogen peroxide decomposing enzyme (H₂O₂) was measured by the potassium permanganate titration method (Borràs-Brull et al., 2021). Glutamine synthetase (GS) was determined using visible spectrophotometry (Haghighat, 2005).

### 2.4 Sequencing and bioinformatics analysis

#### 2.4.1 DNA extraction

Genomic DNA was extracted from soil microbial communities using the E.Z.N.A.^®^ Soil DNA Kit (Omega Bio-tek, Norcross, GA, United States), following the manufacturer’s instructions. DNA quality was assessed by 1% agarose gel electrophoresis, and DNA concentration and purity were measured using a NanoDrop 2000 spectrophotometer (Thermo Scientific, United States).

#### 2.4.2 PCR amplification and sequencing library construction

The extracted DNA was used as a template, and primers 338F (5′-ACTCCTACGGGAGGCAGCAG-3′) and 806R (5′-GGACTAC HVGGGTWTCTAAT-3′) were used for PCR amplification of the 16S rRNA gene (Liu et al., 2016). Ten nanograms (ng) of DNA were extracted from soil samples for PCR amplification of the V3–V4 region of the 16S rRNA gene. Sequencing was conducted using the Illumina MiSeq PE300 platform. The raw sequencing data were quality controlled using fastp (Chen et al., 2018; https://github.com/OpenGene/fastp, version 0.20.0), and paired-end reads were merged using FLASH (Magoč and Salzberg, 2011; http://www.cbcb.umd.edu/software/flash, version 1.2.7). A total of 22,243,836 high-quality sequences were obtained. To minimize sequencing depth effects on subsequent alpha and beta diversity analyses, all sample sequence numbers were rarefied to 24,845. Sequence rarefaction is recommended to ensure comparability across samples. After rarefaction, the average sequence coverage (Good’s coverage) of each sample remained at 99.09%. OTU-level taxonomic annotation was performed using the RDP Classifier (Wang et al., 2007; http://rdp.cme.msu.edu/, version 2.11) against the SILVA 16S rRNA gene database (v138), with a confidence threshold of 70%, to assign taxonomic classifications. The community composition of each sample was statistically analyzed at various taxonomic levels. The 16S functional prediction analysis was conducted using PICRUSt2 (Douglas et al., 2020; version 2.2.0) software.

### 2.5 Functional and life history trait analysis

The functional potential of bacterial communities was predicted using BugBase and PICRUSt 2 (Wang S. et al., 2022; Zhu et al., 2022). Phenotypic predictions of the bacterial communities were made using BugBase.^1^ Functional characteristics of the bacterial communities were predicted using PICRUSt2,^2^ referencing the KEGG pathway database.^3^ Life history traits were calculated by selecting K-strategist and r-strategist members within the bacterial communities (Shi et al., 2023). K-strategists primarily include Acidobacteriota, Actinobacteriota, and Chloroflexi, while r-strategists are mainly represented by Firmicutes, Gemmatimonadota, and Proteobacteria.

### 2.6 Assembly processes analysis

To classify bacterial community assembly into underlying deterministic and stochastic processes, the β-nearest taxon index (βNTI) was calculated using null model generated through 999 randomizations based on the observed data (OTU table and genetic development tree) (Shi et al., 2023; Jiao et al., 2022). βNTI <−2 and βNTI >2 indicated homogeneous and heterogeneous selection (deterministic processes), respectively. |βNTI| <2 indicated that bacterial community assembly was the dominance of stochastic processes. To quantitatively analyze the mechanisms of bacterial community assembly, phylogenetic bin-based null model analysis (iCAMP) was conducted to further segregate the contribution of ecological processes to bacterial assembly (Ning et al., 2020).

### 2.7 Co-occurrence network analysis and ZiPi analysis

Bacterial co-occurrence network at the OTU level was assessed using the Spearman rank correlation (Shi et al., 2023). Only OTUs with relative abundance >0.1% were used in the analyses. Statistical correlations were identified when Spearman’s *r* > 0.7 or <−0.7, and *p* < 0.05 and were then incorporated into the co-occurrence network construction. The node and edge numbers, average clustering coefficient, average degree and graph density were used to evaluate network complexity in this study. Positive and negative correlations between nodes represented cooperative and competitive behaviors (Wang C. et al., 2024).

For the ZiPi analysis, the igraph package and the NetCoMi package (version 1.0.4) in R were used (Olesen, et al., 2007). Based on the Zi value (within-module connectivity) and Pi value (among-module connectivity) of each node, the network nodes were classified into four categories: (1) module hubs (Zi >2.5, Pi ≤0.62), which occupy important positions within modules but have weak associations with other modules, (2) connectors (Zi >2.5, Pi >0.62), which play a significant role in the functional connectivity of the entire network, (3) within-module non-hubs (Zi ≤2.5, Pi ≤0.62), and (4) module outside connectors (Zi ≤2.5, Pi >0.62; Guimerà and Nunes Amaral, 2005).

### 2.8 Statistical analysis

Basic parameters were visualized using the “ggplot2” package in R (version 4.3.1). Statistical analysis was performed using SPSS 23.0 software (SPSS Inc., Chicago, IL), including one-way analysis of variance (ANOVA). A *p*-value <0.05 was considered statistically significant. One-way ANOVA with Duncan’s test was used to assess differences in soil properties, bacterial diversity, life history strategies, and βNTI among the three treatments. Using UPARSE software (Stackebrandt and Goebel, 1994; Edgar, 2013; http://drive5.com/uparse/, version 7.1), sequences were clustered into OTUs based on 97% similarity. Alpha diversity metrics such as Chao 1 and Shannon index were calculated using mothur software (Schloss et al., 2009; http://www.mothur.org/wiki/Calculators), and inter-group differences in alpha diversity were analyzed using the Wilcoxon rank-sum test; principal coordinate analysis (PCoA) based on Bray–Curtis distance algorithm was used to examine the similarity of microbial community structure between samples, and PERMANOVA non-parametric test was performed to analyze whether there were significant differences in microbial community structure between sample groups. The proportion (%) and number of shared and unique OTUs among the four treatments were visualized using the “VennDiagram” package in R. Heatmap analysis was conducted using the “pheatmap” package in R. βNTI and community assembly processes were calculated using the “NST” and “iCAMP” R packages, respectively. Co-occurrence networks were constructed using the “WGCNA” package in R and visualized with Gephi (version 0.10.1). Mantel test and Pearson analysis were performed using the “linkET” package in R to determine the factors influencing bacterial communities and life history strategies. Partial least squares (PLS) regression was conducted using Simca 14.0 (Umetrics, Umeå, Sweden) to distinguish the relative importance of soil physicochemical properties and bacterial characteristics in bacterial community assembly. The direction and magnitude of each factor’s influence on microbial carbon were analyzed using the PLS standardized coefficients. The variable importance in projection (VIP) values were used to assess the relative importance of these variables, with VIP >1 indicating significant influence (*p* < 0.05).

## 3 Results

### 3.1 Soil bacterial community composition and diversity in different straw return methods

**Figure 1:**
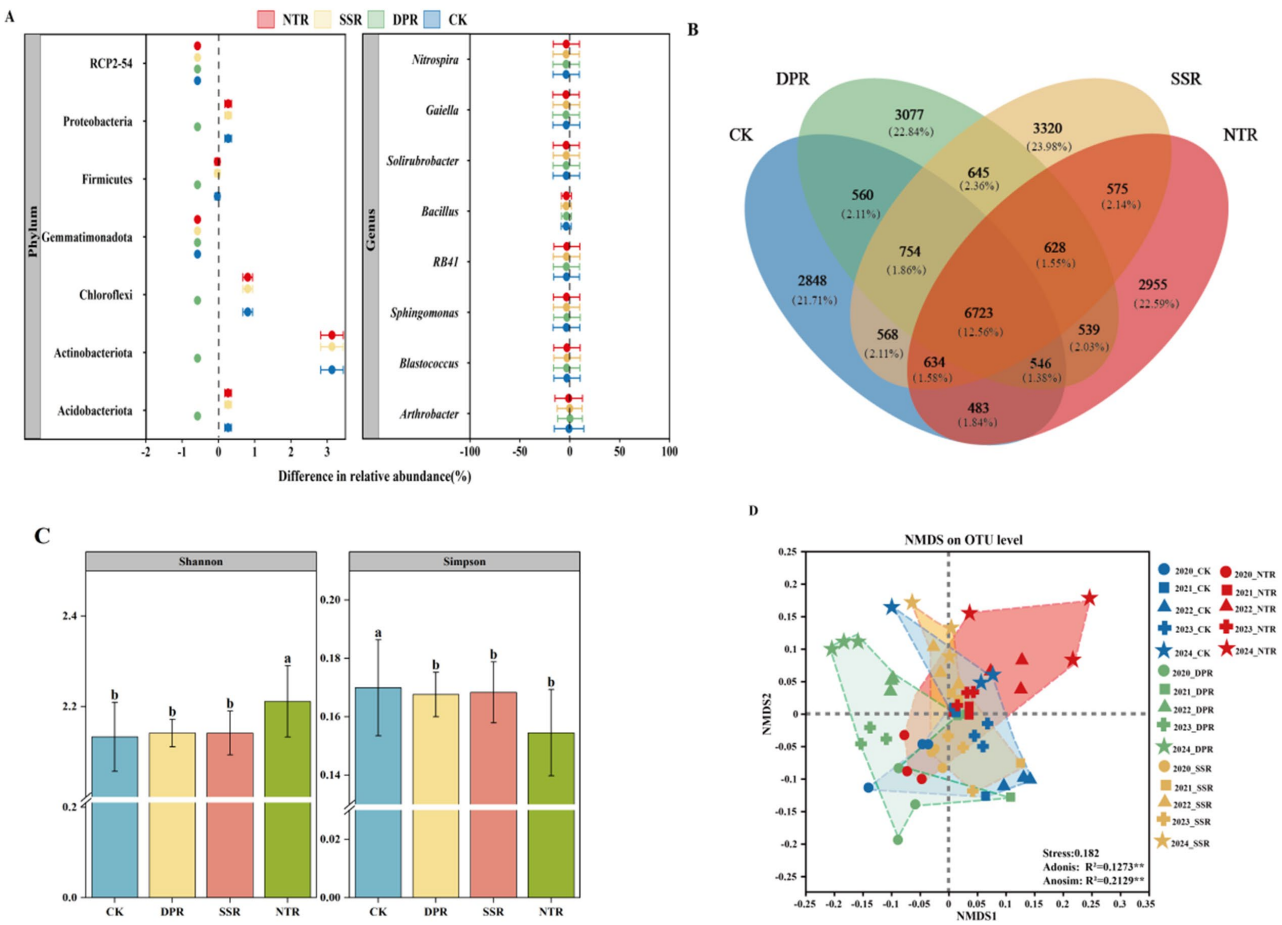
Composition and diversity of soil bacterial communities. **(A)** Differences in relative abundance of soil bacterial communities at phylum and genus levels among different straw-returning practices. **(B)** Venn diagram of bacterial OTUs in CK, DPR, SSR, and NTR treatments. **(C)** Error lines for bacterial α-diversity in CK, DPR, SSR, and NTR treatments are standard deviations (SD) from the mean. Different lowercase letters indicate significant differences among the three treatments (*p* < 0.05). **(D)** Non-metric multidimensional scaled ordination plots of loam bacterial communities for CK, DPR, SSR, and NTR treatments were tested for significance using Adonis and Anosim tests (^**^p < 0.01). CK, farmer’s shallow rotary; DPR, deep rotary straw return; SSR, straw incorporated with subsoiling, and NTR, no-tillage mulching straw return.

The main bacterial phyla identified in each treatment were Acidobacteriota, Actinobacteriota, Chloroflexi, Gemmatimonadota, Firmicutes, and Proteobacteria (Supplementary Figure S2). The relative abundance of these phyla varied significantly across treatments (Figure 1A). Compared with other treatments, the relative abundance of r-strategist bacteria was the highest in the DPR treatment. At the phylum level, Proteobacteria showed a noticeable increase under the DPR treatment (*p* < 0.05). At the genus level, *Nitrosospira* was the most prominent under the DPR treatment (Supplementary Figure S3). In contrast, K-strategy organisms, such as Actinobacteriota, Chloroflexi, Arthrobacter, and Blastococcus, were less abundant in the DPR treatment, with Blastococcus showing the largest difference, being notably lower by 79.93%. Under SSR and NTR treatments, these organisms also declined, though to a lesser extent. Conversely, r-strategy organisms, including *Bacillus*, *Proteobacteria*, *Sphingomonas*, and *Nitrospira*, showed minimal reductions or remained more stable across all treatments, particularly in SSR and NTR, indicating higher resilience. In the CK treatment, the abundance of species remained stable, with K-strategy organisms more abundant than r-strategy organisms. Under DPR treatment, r-strategy organisms, such as *Bacillus* and *Nitrospira,* showed an increase, with *Bacillus* rising by 92.17%, while Firmicutes decreased by 182.31%. Over time, r-strategy organisms steadily increased, while K-strategy organisms declined. In SSR and NTR treatments, r-strategy organisms showed a more gradual increase or remained stable, while K-strategy genera, such as Actinobacteriota and Chloroflexi, declined, particularly after 2022.

The Venn diagram revealed that the number of core OTUs shared among CK, DPR, SSR, and NTR treatments was 6,723 (12.54% of the total), indicating strong environmental adaptation of these core flora (Figure 1B). The number of unique OTUs varied across treatments. The SSR treatment had the highest number of unique OTUs (3,320, 13.88%), followed by DPR (3,077, 12.84%) and NTR (2,955, 12.35%). The CK treatment had 2,848 unique OTUs (11.71%).

The different treatments significantly affected the α-diversity and β-diversity of soil bacterial communities under long-term straw-returning conditions (Figures 1C,D). Simpson’s index showed higher homogeneity in the CK treatment than in the other treatments (*p* < 0.05). No significant differences were observed among the DPR, SSR, and NTR treatments (*p* > 0.05), all showing low homogeneity. Shannon’s index indicated that the NTR treatment significantly increased bacterial diversity, while no significant differences were found between the other treatments (Figure 1C, *p* < 0.05).

In the β-diversity analysis, the NMDS ordination revealed significant segregation of soil bacterial community structures among the different treatments (Stress value = 0.182). The NTR treatment had the widest distribution of points, which were significantly separated from the other treatments. These differences were confirmed by Adonis (*R*^2^ = 0.12, *p* < 0.01) and Anosim (*R*^2^ = 0.21, *p* < 0.01) tests. The community structure of the CK and DPR treatments was closer, while the community structure of the SSR treatment was positioned between CK and NTR (Figure 1D).

### 3.2 Functional and life history characteristics of soil bacteria in different straw return methods

Functional attributes of bacterial communities were predicted by PICRUSt 2 (Supplementary Figure S4). Bacterial communities in the DPR and NTR treatments showed high collaborative metabolic functions, particularly in global metabolic regulation, carbohydrate metabolism and amino acid metabolism pathways, whereas the CK treatment was dominant in specific pathways. The CK treatment showed dominance in specific pathways, such as lipid metabolism, while the SSR treatment had slightly less diversity in metabolic functions but was prominent in some specific functions.

**Figure 2:**
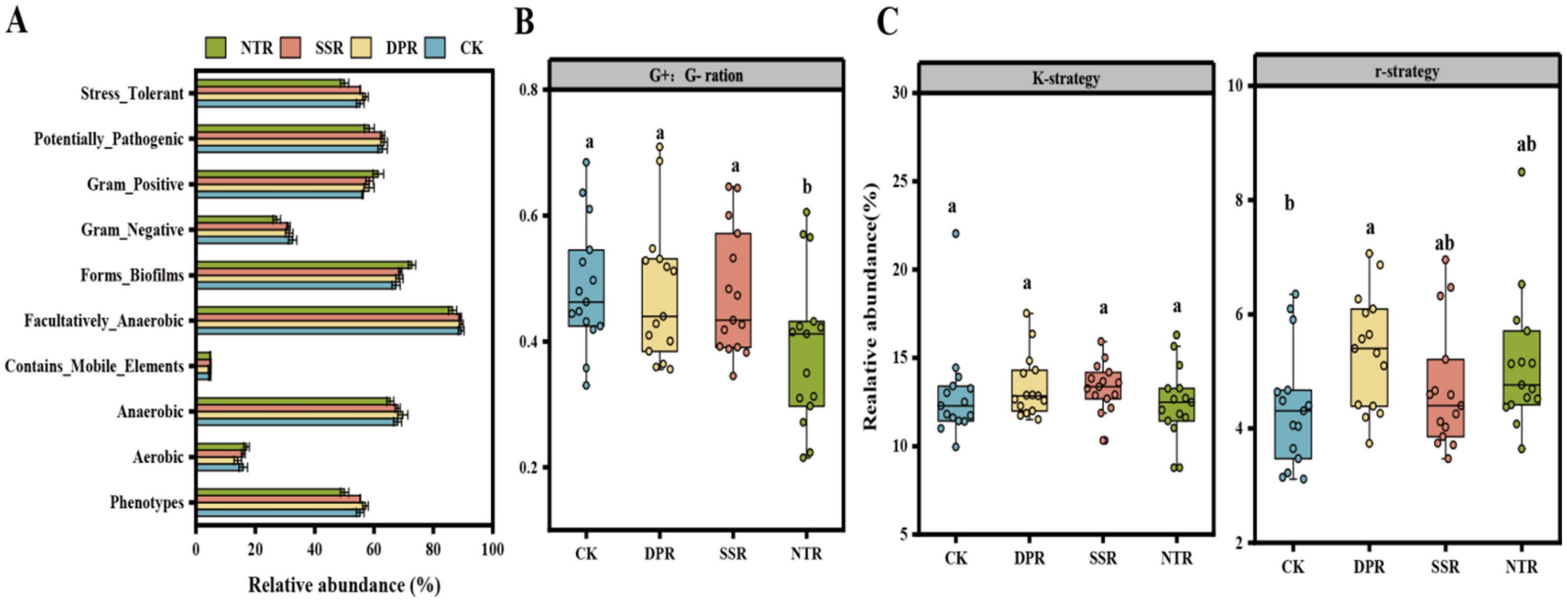
Soil bacterial phenotypes and life history characteristics. **(A)** Bacterial community prediction phenotypes obtained with Bugbase in CK, DPR, SSR, and NTR treatments. **(B)** G+:G− ratios in CK, DPR, SSR, and NTR soils. **(C)** Relative abundance of K-strategy and r-strategy bacteria in CK, DPR, SSR, and NTR soils. The error line is the standard deviation (SD) of the mean. Different lowercase letters indicate significant differences (*p* < 0.05) among the three treatments. G+:G− ratio: phenotypic ratio of Gram-positive to Gram-negative bacteria, K-strategy: relative abundance of representative K-strategy clades (Actinobacteriota, Chloroflexi, and Acidobacteriota), r-strategy: relative abundance of representative r-strategy clades (Proteobacteria, Firmicutes, and Gemmatimonadota) relative abundance.

Bugbase predictions showed that different straw return treatments affected the phenotypic characteristics of bacterial communities (Figure 2A). The NTR and DPR treatments favored the growth of adversity-tolerant, biofilm-forming, and anaerobic bacteria. The CK and SSR treatments favored the colonization of Gram-negative and aerobic bacteria. No significant differences were observed in potentially pathogenic bacteria or mobile genetic element bacteria among the treatments.

The ratio of Gram-positive to Gram-negative bacteria differed significantly (*p* < 0.05) among the CK, DPR, SSR, and NTR treatments, with the NTR treatment showing the lowest G+/G− ratio (0.388), significantly lower than the CK (0.486), DPR (0.478), and SSR (0.475) treatments. Overall, the CK, DPR, and SSR treatments favored the growth of Gram-positive bacteria, while the NTR treatment promoted the dominance of Gram-negative bacteria (Figure 2B).

Among the different treatments, the DPR treatment had the best relative abundance of K-strategists (13.36%) and r-strategists (5.36%), where the abundance of r-strategists was significantly increased by 0.99 percentage points over the CK treatment (*p* < 0.05), the NTR treatment was the second most effective in promoting r-strategy bacteria (5.14%), but less favorable to K-strategy bacteria (12.43%), CK and SSR treatments were closer in promoting K-strategy bacteria (12.92 and 13.34%), but relatively weaker in promoting r-strategy bacteria (4.37 and 4.69%). Overall, DPR treatment most significantly enhanced the synergistic development of K-strategy and r-strategy bacteria, while NTR treatment was more inclined to promote the expansion of r-strategy bacteria.

### 3.3 Soil bacterial assembly process and network analysis in different straw return methods

**Figure 3:**
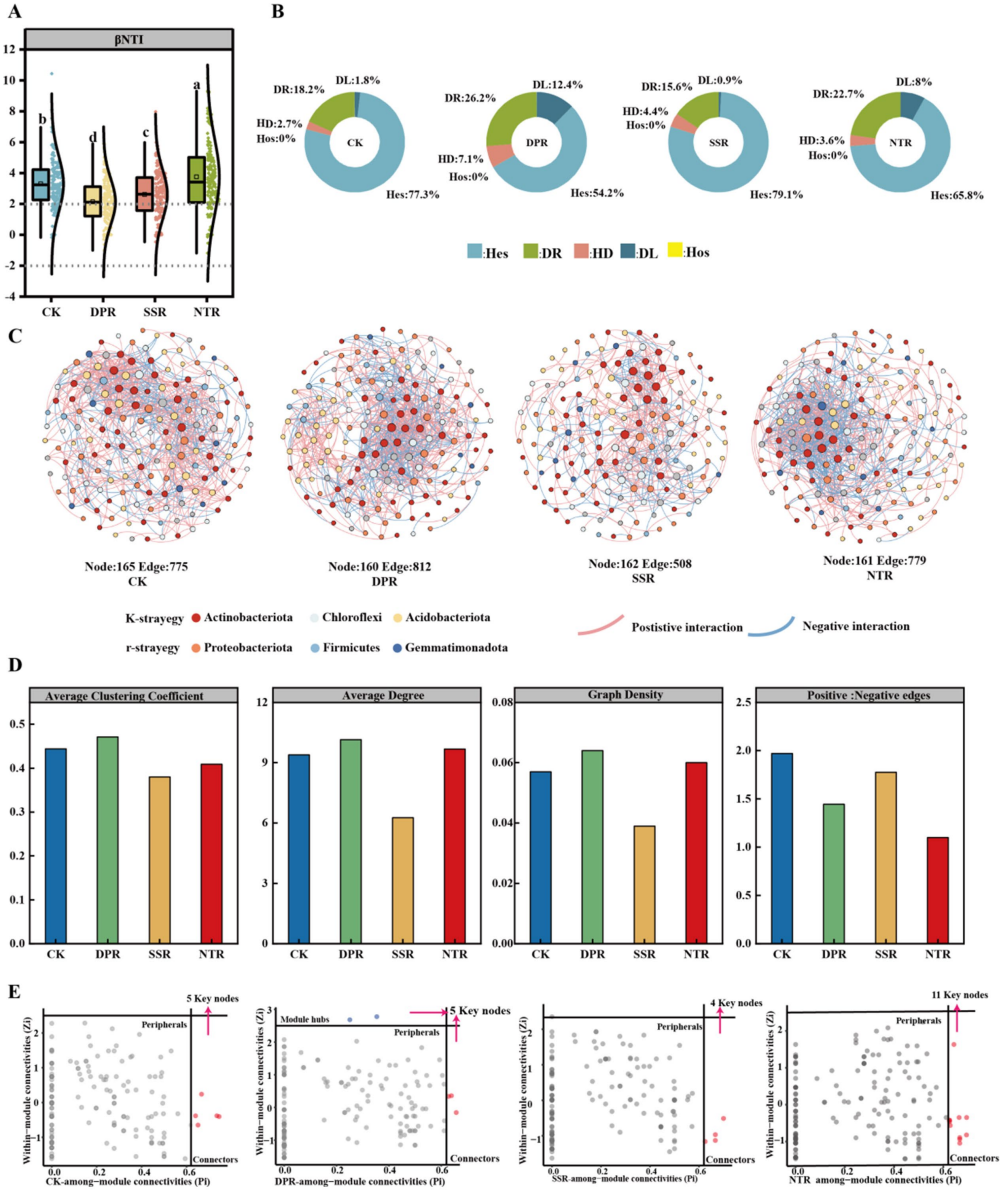
Assembly processes and symbiotic networks of soil bacterial communities. **(A)** β-Nearest taxonomy index (βNTI) of CK, DPR, SSR, and NTR soil bacteria with different lower-case letters indicate significant differences among the three treatments (*p* < 0.05). **(B)** Relative contribution of deterministic and stochastic assembly processes in MM, MP, and MS soils. **(C)** Symbiotic network nodes of bacterial communities in MM, MP, and MS soils indicate individual operational taxonomic units (OTUs), while edges indicate significant correlations between OTUs. The colors of the nodes indicate the main gates of K-strategy and r-strategy, and gray nodes indicate other gates. The size of the nodes is proportional to the number of connections. Only nodes that are significantly (*p* < 0.05) and strongly correlated (Spearman correlation coefficient >0.5 or <−0.5) are connected (edges). The thickness of the edge between two nodes is proportional to the value of the Spearman correlation coefficient. Pink and blue edges indicate positive and negative interactions between two individual nodes, respectively. **(D)** Network statistics including average clustering coefficient, average degree, graph density and ratio of positive and negative edges. **(E)** ZiPi analysis to filter out key nodes. Hes, heterogeneous selection; HoS, homogeneous selection; DL, diffusion limit; HD, homogeneous dispersal; DR, drift.

The results of the null model analysis showed significant differences in the dominant mechanisms of community construction among the different straw-returning methods (Figure 3A; *p* < 0.05). Heterogeneous selection (Hes) was dominant in all treatments, with the proportion ranging from 54.22% (DPR) to 79.11% (SSR). The proportion of stochastic processes varied significantly across treatments (*p* < 0.05). The DPR treatment had the highest proportion of stochastic processes (38.66%), with drift (DL) and diffusion restricted (DR) contributing 12.44 and 26.22%, respectively. The SSR treatment had the lowest proportion of stochastic processes (16.45%), with drift accounting for 0.89%, while heterogeneous selection was 79.11%. CK and NTR treatments were intermediate, with shallow tillage biased towards deterministic processes, while NTR enhanced stochastic processes due to lower soil disturbance. SSR and CK treatments were dominated by deterministic processes, with SSR contributing most to a stable microbial community. NTR treatment increased the contribution of stochastic processes while maintaining soil stability (Figure 3B).

The co-occurrence network showed that the DPR treatment had the highest ecological network complexity, with 169 nodes, 812 edges, 59.11% positive interactions, and a mean degree of 10.15 (Figures 3C,D). The K-strategy bacteria (e.g., Actinobacteriota, Chloroflexi) had a relative abundance of 60.62%, while r-strategy bacteria (e.g., Proteobacteria, Firmicutes) had a relative abundance of 29.99%. The NTR treatment had 161 nodes, 779 edges, 52.37% positive interactions, and a mean degree of 9.677. The relative abundance of K-strategy bacteria was 58.79%, and r-strategy bacteria had a relative abundance of 28.58%. The CK treatment had 165 nodes, 775 edges, 66.32% positive interactions, and a mean degree of 9.394. The relative abundance of K-strategy bacteria was 62.61%, and r-strategy bacteria had a relative abundance of 29.7%. The SSR treatment had 162 nodes, 508 edges, 63.98% positive interactions, a mean degree of 6.272, and the lowest relative abundance of K-strategy bacteria at 58.64%, while the relative abundance of r-strategy bacteria was the highest at 30.87%.

ZiPi analysis showed that the DPR treatment exhibited the highest degree of modularity and network complexity (two modular hubs, three connectors), was dominated by K-strategy bacteria, and had the best ecological function and stability. The NTR treatment had the highest number of key nodes (11 connectors), with high colony diversity and network collaboration, and balanced functionality, and the CK had a key node number of five connectors, a dominated by K-strategy bacteria, with moderate stability but low complexity. The SSR treatment, with a minimum of four connectors of key nodes, was dominated by r-strategy bacteria, with the lowest degree of modularity, and the worst collaboration and network stability (Figure 3E).

### 3.4 Factors influencing soil bacterial community in different straw return methods

**Figure 4:**
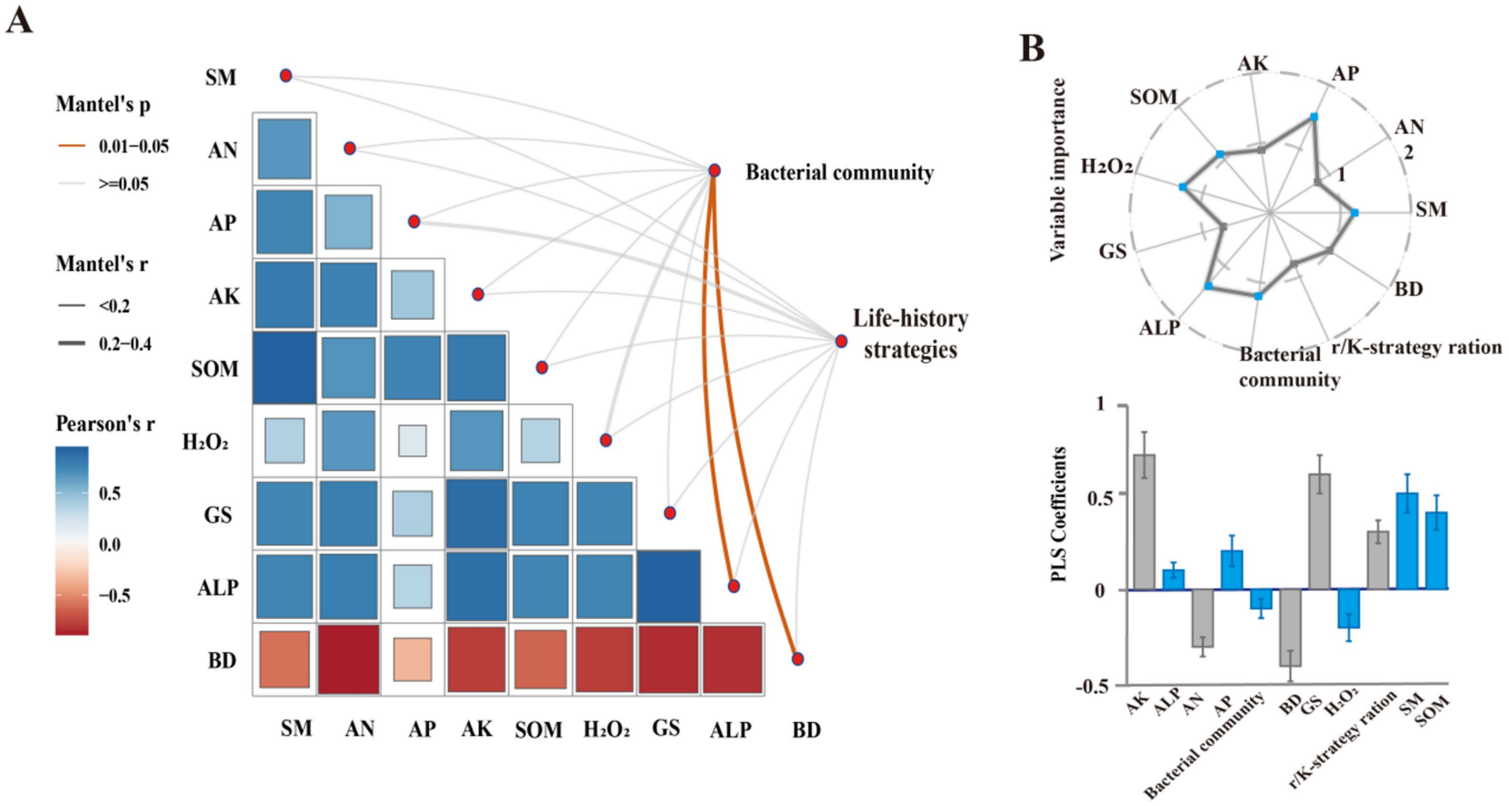
Drivers of bacterial community composition, life history and assembly processes. **(A)** Relationships between bacterial communities, life history strategies and soil properties based on Pearson’s correlation and Mantel test. **(B)** Relative importance of soil physicochemical and microbiological properties on bacterial assembly processes in CK, DPR, SSR, and NTR treatments. The upper panel shows the variable predicted importance (VIP) values and the lower panel shows the partial least squares (PLS) standardized coefficients (±SEM). The error line is the standard deviation (SD) of the mean. Higher VIP values indicate greater importance in determining the assembly process. Blue dots indicate VIP values greater than 1. PLS standardized coefficients show the direction and magnitude of each variable’s impact on the assembly process. Variables with blue bars are significant (*p* < 0.05) where the VIP value is greater than 1. System status: BD, soil bulk density; SM, soil moisture content; AN, soil dissolved alkaline nitrogen; SOM, soil organic matter; AP, soil quick phosphorus; ALP, alkaline phosphatase; Glu, glutamine synthetase; H_2_O_2_, catalase.

Mantel analysis (Figure 4A) showed that soil physicochemical and microbial characteristics significantly influenced soil bacterial community composition and life history strategies in CK, DPR, SSR, and NTR treatments. Soil bacterial community composition was significantly positively correlated with ALP and significantly negatively correlated with BD (*p* < 0.05). Partial least squares (PLS) analysis showed that bacterial assembly processes in the CK, DPR, SSR, and NTR treatments were significantly controlled by AP, SM, ALP, H_2_O_2_, SOM, and bacterial community composition (*p* < 0.05, VIP >1; Figure 4B).

## 4 Discussion

### 4.1 Effects of different straw return methods on the composition, diversity and structure of soil bacterial communities over the years

Soil bacteria are indispensable in regulating ecological processes and functions. Annual straw fertilization can alleviate the pressure on the soil environment and promote the development of the microbiota (Chu et al., 2024). In this study, the relative abundance of major phyla and genera differed between straw-returning conditions in successive years, as did the major bacteria employing r- and K-strategies (Figures 1A, 2C; Supplementary Figures S2, S3, *p* < 0.05). Years can influence soil bacterial community composition in semi-arid cropping systems (Ouverson et al., 2021). Compared to CK, DPR treatment, r-strategy bacteria such as Firmicutes and Proteobacteria and related genera such as *Bacillus* and *Nitrospira* showed stronger adaptation and the relative abundance of *Bacillus* increased by 92.17%. However, K-strategy bacteria such as Actinobacteriota and Chloroflexi and their related genera (e.g., *Arthrobacter* and *Blastococcus*) were significantly reduced, with the abundance of *Blastococcus* decreasing by as much as 79.93%. This suggests that DPR treatment promoted the rapid multiplication and adaptive capacity of r-strategy bacteria (e.g., *Bacillus*) and inhibited the growth of K-strategy bacteria (e.g., *Blastococcus*) by changing the physicochemical properties of the soil (e.g., soil bulkiness, moisture, and nutrient content) due to the fact that the r-strategy bacteria have a greater environmental adaptability and rapid response ability to unstable environment (Yang et al., 2020). It indicated the selective effects of different straw treatments on the life history strategies and functional potential of soil bacterial communities. In addition, successive years of DPR treatments improved nutrient uptake and microbial diversity in corn. Simpson’s index showed that soil bacterial community homogeneity was significantly higher in the CK treatment than in the other treatments, suggesting that the community was more homogeneous under the CK treatment (*p* < 0.05). In contrast, the community homogeneity of DPR, SSR and NTR treatments was lower and the difference was not significant. This result supports the theory of Li H.-Z. et al. (2022) that soil management practices may exert selective pressure on bacterial communities, especially under conditions of nutrient excess or environmental instability, where certain bacterial taxa may be dominant, leading to a decrease in community homogeneity. This finding is consistent with Schimel and Schaeffer (2012) who noted that proper nutrient management can support diverse microbial communities and enhance soil ecosystem stability. The increased diversity of the NTR treatment may be due to specific nutrient conditions that promote the survival of more bacterial taxa or reduce environmental competitive pressures, thus providing space for more species to survive. Changes in microbial diversity may depend on soil management strategies, especially nutrient management, under different straw-returning practices, which can lead to healthier and more efficient soil microbial communities, thereby improving soil quality and crop growth (Chen et al., 2021). For example, the core OTUs shared among CK, DPR, SSR and NTR treatments were 6,723, which accounted for 12.54% of the total number of OTUs, suggesting that these core colonies have strong environmental adaptability. In contrast, the number of unique OTUs in the CK treatment was 2,848 (11.71%), which was slightly lower than the other treatments (Figure 1B). These results further demonstrated the different roles of different straw return treatments in microbial community reorganization and selectivity (Song et al., 2020). This study reveals the different responses of soil bacteria to different straw-returning methods in successive years.

In the β-diversity analysis, the NMDS ordination showed that the soil bacterial community structures of different treatments were significantly separated, with the NTR treatment having the most distinctive community structure and being significantly separated from the other treatments (Figure 1D, *p* < 0.05). The significance of this difference was also verified by the results of Adonis and Anosim tests. Overall, the CK treatment maintained high community evenness, the NTR treatment significantly increased community diversity and significantly altered community structure, while the SSR and DPR treatments had relatively little effect. It showed that different straw return methods in successive years led to significant differences in soil bacterial community structure (Li Y. et al., 2022). Soil extracellular enzyme activities were also affected (Yang et al., 2022), especially GS and ALP in DPR soils and H_2_O_2_ in NTR soils (Supplementary Table S3). In addition, DPR and NTR treatments (with higher average clustering coefficients and graph densities) had higher network complexity (Figure 3D), which is supported by the fact that successive years of DPR and NTR have enhanced microbial interactions between microorganisms (Xu et al., 2023). Moreover, in semi-arid cropping systems, different tillage practices combined with straw return stimulate soil microbial biomass and provide more opportunities for bacterial interactions (Bu et al., 2020), given that bacterial network complexity can also be driven by bacterial r-/K-strategy ratios (Wang C. et al., 2024), soil bacterial network complexity in successive DPR and NTR treatments increased with the r-strategy bacterial ratio increased (Figure 3C and Supplementary Table S4). In addition, the higher the ratio of negative correlation, the smaller the perturbation, which promotes the stability of bacterial network (Shi et al., 2021). The network of bacteria in the DPR treatment was more stable, with a higher mean degree (10.15) and positive connectivity percentage (59.11%). While CK treatment showed opposite trend in terms of network stability, Yang et al. (2016) indicated that under drought conditions, straw return produced less stable bacterial network and more stable fungal network. Therefore, DPR treatment can regulate the sustainability of agriculture under drought conditions through soil microbial response.

### 4.2 Effects of different straw-returning methods on soil bacterial community structure and function in successive years

In this study, compared with non-returned (CK), straw return significantly enhanced the dominant role of homogenous selection and drift (Figure 3B), especially in the treatments with more organic matter return (e.g., the group of complete straw return). This phenomenon is closely related to resource availability, especially changes in organic matter and nutrient content (e.g., nitrogen and phosphorus content) in the soil that may have facilitated selective microbial acclimation and remodeling of community structure (Liu L. et al., 2024). Differences in acclimatization led to ecological niche partitioning under environmental heterogeneity (Lin et al., 2021), whereas the effects of different straw-returning practices over successive years could shape bacterial symbiosis and community assembly depending on the βNTI values (Figure 3A). Compared with NTR, the CK treatment had a greater deterministic process with significantly lower βNTI values, which is a common phenomenon under relatively low resource conditions (Hao et al., 2023). For example, the bacterial community under low-resource conditions showed stronger deterministic processes in soils with successive years of straw return, which may be related to the gradual accumulation of soil organic matter (Yang et al., 2021). To some extent this bacterial assembly process was related to the higher nutrient content in the DPR treatment (Supplementary Table S1). Differences in bacterial communities in CK, DPR, SSR, and NTR soils may be attributed to the assembly process, particularly as a result of diffusion limitation at intermediate or short geographic distance differences (Liu, 2023). Thus, DPR and NTR treatments increased the contribution of diffusion limitation to stochastic processes and played a key role in the assembly of soil bacterial communities.

Bacterial phenotypes and related functions of soil were altered to different degrees by different straw-returning practices over the years. The diversity and functional stability of microbial metabolism can be enhanced by stimulating the interactions of bacterial communities under different straw-return practices (Liu X. et al., 2023). Straw return not only affected the composition of the bacterial community, but also significantly altered the bacterial phenotype and tolerance (Wang X. et al., 2022). DPR treatment increased the relative abundance of anaerobic bacteria higher, while SSR treatment significantly increased the abundance of aerobic bacteria. This change may be related to the changes in oxygen supply in the soil after straw return. Siedt et al. (2021) showed that straw return improves soil aeration, which affects microbial life history strategies. In addition, by modulating microbial life-history strategies, straw return increased tolerance to environmental stresses (e.g., hypoxia, changes in pH), which further contributed to the growth of beneficial microorganisms in the soil (Chen et al., 2024).

PICRUSt 2 software was used to analyze the effects of different straw-returning methods on the functional profiles of soil bacterial communities in successive years. Metabolic pathways at the first and second levels showed similarity among different straw return treatments (Supplementary Figure S2). In general, taxonomic diversity and metabolic complexity of microbial communities are related to life history strategies and dependent on resource availability (Lauber et al., 2008). In this study, the relative abundance of genes associated with Meta function was the greatest, especially in the global and overview maps. Moreover, key metabolic pathways such as amino acid metabolism and carbohydrate metabolism were enhanced under DPR and SSR treatments, suggesting that straw return may have promoted the metabolic activity of microorganisms in the soil by providing more organic carbon sources (Shah et al., 2024). Although there were differences in the specific effects of different straw-return treatments on metabolic pathways, it was shown that straw-return promoted the adaptability and tolerance of soil bacterial communities in most cases by enhancing the functional stability of soil microorganisms (Zhang Y. et al., 2024), further supporting the sustainability of soil ecosystems.

ZiPi analysis showed significant differences in the degree of network modularity, complexity, and collaboration among the different treatments (Figure 3E, *p* < 0.05). DPR exhibited the highest degree of modularity and network complexity (two modular hubs, three connectors) and was dominated by K-strategy bacteria, which significantly optimized mycorrhizal collaborative and ecological stability, and is an ideal management practice to enhance soil health and ecological function (Wang et al., 2023). NTR had the highest number of key nodes (11 connectors), which significantly enhanced mycorrhizal diversity and network collaboration, and exhibited good ecological balance and functional stability (Xu et al., 2023). In contrast, the CK treatment had limited network complexity and functional optimization, and weak collaboration despite maintaining the basic functions of the flora, the deep straw return (SSR) treatment had the lowest degree of modularity and complexity, was dominated by r-strategic bacteria, and had enhanced flora competitiveness but poorer collaboration, and limited ecological functions (Khan et al., 2023). Therefore, DPR and NTR have significant advantages in optimizing soil microbial network structure and enhancing soil health, while CK and SSR need to be further optimized in combination with other measures.

### 4.3 Study on the driving factors of soil bacterial community in different straw return methods in successive years

Changes in biotic and abiotic factors induced by different straw-returning practices control the soil microbial community’s response to bacteria, and the soil microbial community is affected by multiple and complex factors that modify the soil habitat (Liu Q. et al., 2024). Similar environmental conditions greatly increase the similarity of soil bacterial communities (Belykh et al., 2024). The composition of soil bacterial communities was significantly positively correlated with ALP and significantly negatively correlated with BD (*p* < 0.05). Elevated ALP was closely associated with enhanced metabolic functions of bacterial communities, which may be attributed to the fact that alkaline phosphatase supports microbial growth and ecological functions by facilitating the release of soil phosphorus, which provides the bacterial community with an abundant source of usable phosphorus (RAES) (Raiesi and Salek-Gilani, 2018). In contrast, higher soil bulk density (BD) suppressed bacterial community diversity and functional activity by limiting soil porosity and water retention capacity (Fang et al., 2024). PLS analysis further revealed that AP, SM, ALP, H_2_O_2_, and SOC were identified as key driving variables. These factors directly regulate the diversity and life history strategies of bacterial communities by providing sources of organic matter and nutrients (Wang Q. et al., 2024). SOC is a major carbon source for microorganisms, and was significantly elevated in DPR, providing an energy base for r-strategy bacteria (e.g., Proteobacteria and Firmicutes) to rapidly colonize (Zhan et al., 2024). Furthermore, Ma et al. (2022) revealed that biological interactions predicted bacterial communities better than environmental factors, thus driving the assembly of soil bacterial communities in arid ecosystems. Keystone taxa and interactions between bacteria and fungi were significantly associated with community assembly (Shi et al., 2023). An addition, enzymes (ALP and H_2_O_2_), as biotic factors, influenced the life history strategies of bacteria in CK, DPR, SSR, and NTR soils. There were subtle differences in the composition, diversity and other characteristics of soil bacterial communities in different straw return treatments. In addition, the elevated AP created a more favorable environment for phosphorus metabolism-associated bacteria [e.g., phosphorus solubilizing bacteria (PSB)] to survive, which promoted the ecological functions of K-strategy bacteria (e.g., Actinobacteria and Chloroflexi) (Liu L. et al., 2023). Therefore, this study focused on the factors affecting the common corn field by different straw return methods in successive years. In order to deeply understand the feedback relationship between different straw return methods and soil microbial communities in successive years, future studies should further combine diversified ecological analysis tools to explore the synergistic effects between straw return and other agricultural measures (e.g., fertilizer application, crop rotation, no-tillage), and deeply analyze the mechanism of long-term straw return on the dynamics of soil microbial communities, so as to provide theoretical support for the sustainable development of agro-ecosystems. Theoretical support for sustainable development of agro-ecosystems.

## 5 Conclusion

Different straw-returning methods in successive years significantly affected the composition, function, assembly mechanism and network properties of soil bacterial communities by modulating soil physicochemical properties (e.g., ALP, BD, SOC) and enzyme activities (*p* < 0.05). Among them, DPR significantly promoted the synergistic development of K-strategy bacteria and r-strategy bacteria to optimize metabolic functions and network stability, while the opposite was true for CK treatment, no-tillage mulching (NTR) increased community diversity, enhanced environmental adaptation (G+/G− ratio of 0.388), and promoted bacterial collaboration, which was suitable for low-disturbance management, although deep straw removal (SSR) had a significant effect on community reorganization, network DPR and NTR treatments were outstanding in function optimization and low-disturbance management, respectively, while SSR treatment promoted community reorganization effectively but did not enhance long-term ecosystem stability. In the future, it is necessary to comprehensively evaluate the comprehensive effects of straw return methods on sustainable agriculture by combining multi-dimensional indicators such as crop yield, soil quality and resistance, and explore the synergistic effects with other management measures in order to promote the efficient and sustainable development of agroecosystems.

## Data Availability

The original contributions presented in the study are publicly available. All raw sequencing data were deposited in the National Center for Biotechnology Information (NCBI) BioProject database, under accession number PRJNA1222571.

## Footnotes

^1^ https://bugbase.cs.umn.edu/index.html

^2^ https://github.com/picrust/picrust2

^3^ http://www.genome.jp/kegg/

## Supplementary material

At the beginning of the experiment, the basic soil fertility in 2020, the significant analysis of the difference in soil physical and chemical properties enzyme activity from 2020 to 2024, the main bacteria, meteorological factors, relative abundance of phyla and genus levels of K-strategies and r-strategies, and the functional properties of soil bacterial communities are displayed in Supplementary material.
